# Multiple Cracks Detection in Pipeline Using Damage Index Matrix Based on Piezoceramic Transducer-Enabled Stress Wave Propagation

**DOI:** 10.3390/s17081812

**Published:** 2017-08-12

**Authors:** Guofeng Du, Qingzhao Kong, Hua Zhou, Haichang Gu

**Affiliations:** 1 School of Urban Construction, Yangtze University, 434023 Jingzhou, China; gfdu@yangtzeu.edu.cn; 2 Department of Mechanical Engineering, University of Houston, Houston, TX 77204, USA; qkong@uh.edu; 3 School of Civil Engineering and Architecture, Wuhan University of Technology, 430072 Wuhan, China; zhouhua@whut.edu.cn

**Keywords:** piezoceramic transducer, stress wave propagation, wavelet packet analysis, pipeline crack detection, multiple cracks detection, structural health monitoring

## Abstract

Cracks in oil and gas pipelines cause leakage which results in property damage, environmental pollution, and even personal injury or loss of lives. In this paper, an active-sensing approach was conducted to identify the crack damage in pipeline structure using a stress wave propagation approach with piezoceramic transducers. A pipeline segment instrumented with five distributed piezoceramic transducers was used as the testing specimen in this research. Four cracks were artificially cut on the specimen, and each crack had six damage cases corresponding to different crack depths. In this way, cracks at different locations with different damage degrees were simulated. In each damage case, one piezoceramic transducer was used as an actuator to generate a stress wave to propagate along the pipeline specimen, and the other piezoceramic transducers were used as sensors to detect the wave responses. To quantitatively evaluate the crack damage status, a wavelet packet-based damage index matrix was developed. Experimental results show that the proposed method can evaluate the crack severity and estimate the crack location in the pipeline structure based on the proposed damage index matrix. The sensitivity of the proposed method decreases with increasing distance between the crack and the mounted piezoceramic transducers.

## 1. Introduction

Pipeline transportation plays an important role in the national economy, and it is the major means of transporting oil and gas. The advantages of pipeline transportation include: (1) large transport volume which can provide a smooth, uninterrupted transport; (2) short construction period and low cost of construction compared to railway transportation; (3) relatively little influence on the environment and climate change when accident occurs; (4) low cost of transport with less energy consumption. Pipeline transportation offers continuous delivery of the energy, and it does not need load stroke and transportation system, which ensures a high transport efficiency. However, cracks occur in the pipeline in service due to corrosion, fatigue, and inappropriate operations, which result in serious consequences including loss of property, personal injury, or even loss of lives and serious ecological pollution. Therefore, detection of the pipeline crack damage in real-time has become an important research topic to ensure the safety of pipeline transportation. The current methods for pipeline crack detection include: the magnetic flux leakage method [1,2], ultrasonic method, eddy current method, ray method [3,4], and magnetic method [5]. The above methods need technicians to operate, and they cannot detect damages in real-time, especially over long distances. The low efficiency and high cost limit the application of the traditional damage detection methods for long-distance pipeline systems. Therefore, a real-time monitoring system is highly in demand for the damage detection of a long-distance pipeline system. 

In recent years, structural health monitoring and non-destructive testing have received significant attention [6,7,8,9,10]. Due to the fast response, low cost, suitability for energy harvesting, and capability for actuation and sensing [11,12,13], PZT (lead zirconate titanate)-based transducers and stress wave-based technique have been increasingly used in the structural health monitoring of various mechanical and civil structures [14,15,16,17]. Siu et al. [18,19] studied the stress wave communication in concrete using piezoceramic transducers. Feng et al. [20,21,22] used stress wave approaches to detect damages in concrete pipe and concrete piles instrumented with embedded smart aggregates. Kong et al. [23,24] implemented stress wave technique for concrete hydration monitoring and water presence detection in concrete cracks. Du et al. [25,26,27] performed experimental studies to detect pipeline crack or corrosion by using PZT-induced stress waves. Hong et al. [28] used piezoceramic transducers to perform active monitoring of a pipeline tapered thread connection. Zhu et al. [29] used piezoelectric sensors to detect gas pipeline leakage. Cheng et al. [30] reviewed stress wave-based pipeline monitoring. Breon et al. [31] studied wall thinning and other damage conditions via ultrasonic tomographic reconstruction of the pipeline structure. Velsor et al. [32] developed the reconstruction algorithm for the probabilistic inspection of damage (RAPID) to construct tomographic images of multiple defects in pipelines. Qing et al. [33] developed a real-time active pipeline integrity detection system by utilizing the SMART Layer technology. The developed system can visualize the approximate location and the extent of corrosion. Corrosion sizing and depth can be quantitatively evaluated. Bergman et al. [34] developed a real-time active pipeline integrity detection system for the detection, localization, and quantification of corrosion and erosion damage in metal piping. The proposed system consists of a network of miniature ultrasonic sensors embedded in a thin dielectric film that can be integrated with the pipe. Ravanbod [35] introduces fuzzy decision-based neural network algorithms for the detection and classification of corrosions in the pipeline inspection.

In this paper, a feasibility study for the damage identification in pipeline structure was conducted using piezoceramic-based stress wave propagation approach. Five distributed piezoceramic transducers were surface-mounted on a pipeline segment specimen. Four cracks were artificially cut on the specimen, and in the test, each crack had seven damage cases corresponding to different crack depths to simulate crack development. A series of tests were conducted using a guided stress wave propagating between various piezoceramic actuator–sensor pairs. To quantitatively evaluate the damage severity for multiple cracks and demonstrate the damage location information, a wavelet packet-based damage index matrix was developed. The baseline of health status was first obtained, and the signals at damage status was compared with the baseline. Different actuator–sensor pairs were used for the severity evaluation of multiple cracks in the pipeline inspection. The proposed damage index matrix reveals the damage history development of each actuator–sensor pair, and the damage development trends at various locations are visually demonstrated via a three-dimensional damage index matrix plot. Damage index value corresponds to the energy loss in the wave propagation, which correlates with the damage severity. By studying the damage values of the different actuator–sensor pairs in a damage index matrix, the damage severity for the cracks along the wave propagation path is quantitatively evaluated for multiple locations. The damage location information can also be extracted through the proposed damage index matrix. The innovation of the proposed approach is that it detects the multiple cracks of pipeline structure in real-time without using bulky equipment. The proposed approach uses wavelet packet analysis as the signal processing tool, which enables the inspection of relatively narrow frequency bands over a relatively short time window compared with wavelet analysis. The proposed health monitoring approach will be more sensitive to detect cracks by using a wavelet packet-based damage index matrix. To verify the effectiveness of the proposed damaged index matrix in the pipeline integrity evaluation, an experiment was performed on a seamless steel pipeline segment specimen with multiple man-made cracks. Experimental results show that the proposed damage index matrix visualizes the damage severity for various actuator–sensor pairs at different locations for multiple cracks. The experimental results also demonstrate that the sensitivity of the proposed method decreases with the increase of the distance between the crack and the mounted piezoceramic transducers.

## 2. Principles

**Figure 1:**
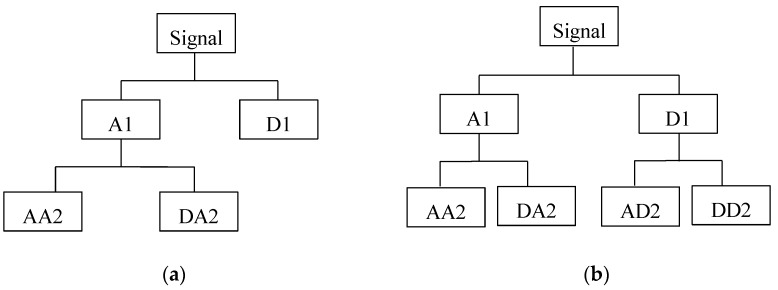
Comparison of wavelet decomposition tree and wavelet packet decomposition tree. (**a**) Wavelet decomposition tree at level 2; (**b**) Wavelet packet decomposition tree at level 2.

In recent decades, various signal-processing methods have been explored to analyze the stress wave information and quantify the damage severity. These methods include spectrum analysis, inverse spectrum analysis and statistical analysis; inverse mapping theory, neural network theory, reconstitution theory, and homomorphism analysis technology [36,37,38,39]. The main object of these methods is to quantify the energy dissipation value of the received signal. Wavelet transform is an effective signal processing approach to analyze the sensor signal for pipelines with cracks [40,41,42]. Compared with wavelet analysis, wavelet packet analysis has the advantage that it enables the inspection of relatively narrow frequency bands over a relatively short time window. In wavelet analysis, a signal is split into an approximation and a detail. The approximation itself is split into a second-level approximation and detail. This process is repeated as shown in Figure 1a. However, in the wavelet packet analysis, the detail as well as the approximation is split into the next level approximation and detail, as shown in Figure 1b.

In this paper, a wavelet packet-based damage index matrix is developed to evaluate the damage severity and estimate the crack location. The main procedures to compute the damage index matrix are as follows. The received signal *X* from one measurement is decomposed into *n*th-level wavelet packet decompositions which are 2*^n^* signal subsets {*X*_1_, *X*_2_, *…*, *X*_2_*^n^*} [25]. In each signal subset *X_j_*, (*j* = 1, 2, …, 2*^n^*), *X_j_* can be expressed as

*X_j_* = [*X_j_*_,1_, *X_j_*_,2_, …, *X_j,m_*]

where *m* is the number of the sampling data.

The energy of each signal subset *E_j_* is computed using the following equation.

$$E_{j}=\;\sum_{k\;=\;1}^{k\;=\;m}X_{j,k}^{2}$$

The damage index is defined by using root-mean-square deviation (RMSD), which is given by

$$I=\sqrt{\frac{\sum_{j=1}^{2^{n}}{\left(E_{i,j}-E_{0,j}\right)}^{2}}{\sum_{j=1}^{2^{n}}E_{0,j}^{2}}}$$

where i is time index corresponding to different measurements; $E_{0,j}$ refers to energy of the *jth* subset signal for the structure under health condition; and $E_{i,j}$ refers to the energy of the *jth* subset signal for the structure under damage with *ith* time index. 

The proposed damage index matrix *M* is defined as

*M*_m×n_ = [*I_a_*_,*b*_]

where the matrix element *I_a_*_,*b*_ represents the damage index associated with the *a*th actuator–sensor pair at the *b*th damage case (*a* = 1, 2, …, m and *b* = 1, 2, …, n; m is total number of chosen actuator–sensor pairs; n is the total number of chosen damage cases). In this paper, Daubechies 5 wavelet (db5) is used as a mother wavelet, and decomposition level is chosen as 5. The damage index value demonstrates the energy attenuation ratio due to the crack existence in pipeline. The greater the damage index value is; the more energy is attenuated by the crack, which means the more severe the damage status.

The wavelength needs to be small in order to be sensitive enough to detect the crack, which means a high-frequency excitation is needed. Power plays a major role in transmission distance. For a large-scale structure, a high-frequency excitation with high power is needed for better accurate health monitoring results.

## 3. Experimental Setup

### 3.1. Pipeline Specimen

**Figure 2:**
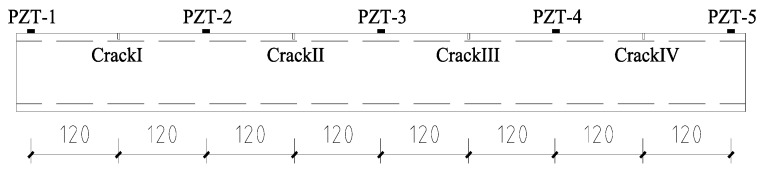
Drawings of the pipeline specimen (unit: mm).

The pipeline segment specimen was made of Q235 seamless steel. The length of the specimen was 1 m. The outer diameter was 100 mm and the inner diameter was 80 mm, the wall thickness of the pipe wall was 10 mm. Four artificial cracks in the pipeline were sequentially cut, and each crack was cut under six operating conditions (OCs). The widths of all the cracks were the same: 0.5 mm. A total of twenty-four damage cases (operating conditions) are listed in Table 1. Five piezoceramic transducers were surface-mounted on the pipeline specimen using epoxy resin. Epoxy resin will obtain high strength and stiffness after it cures, and it will not affect stress wave propagation. The five piezoceramic transducers were distributed evenly along a straight line on the pipeline with 240-mm between two adjacent transducers. The type of the piezoceramic transducer was lead zirconate titanate-5H (PZT-5H). Properties of the PZT-5H are shown in Table 2. The pipeline dimension, crack location, and sensor location are shown in Figure 2.

### 3.2. Experimental Setup and Procedures

**Figure 3:**
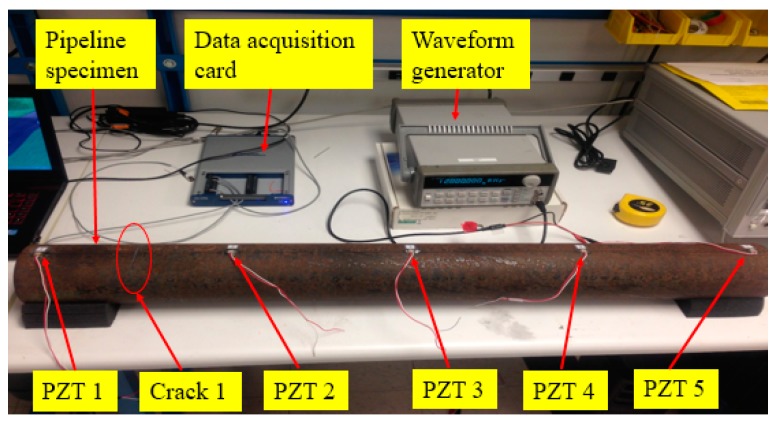
The experimental setup.

The experimental setup included the pipeline segment specimen, an arbitrary waveform generator (Agilent 33120A), a data acquisition card (NIUSB-6363), and a computer, as shown in Figure 3. A sweep sine wave signal was programmed by the waveform generator and used as the excitation source for the selected PZT actuator. The sweep sine signal had an amplitude of 10 V and the frequency was changed from 60 kHz to 200 kHz in one second. According to the designed order of the crack appearance (from Crack 1 to Crack 4) under each operating condition, one of the five transducers was selected as an actuator, and the other four transducers were used as sensors to detect the wave responses. Different actuator–sensor pairs were used to evaluate the damage status at different locations. By switching the actuator–sensor pair orders or sequences, the damage status at different locations was evaluated. Another advantage of piezoceramic transducers is that the transducer can function as either an actuator or a sensor, which provides more options of actuator–sensor pairs. To avoid the wave interference by multiple actuation sources at the same time, the proposed approach uses one actuator each time and then rotates the actuator sequence. For example, in the series of tests regarding the first crack, PZT-1 was firstly used as the actuator, while PZT-2, PZT-3, PZT-4, and PZT-5 were used as the sensors to detect the stress wave responses under OC1. Then, the PTZ-2 was chosen as the actuator, while PZT-1, PZT-3, PZT-4, and PZT-5 were used as the sensors. The rest of the tests under OC1 followed the same criteria until every PZT transducer had been selected as an actuator for test. A total of twenty-four operating conditions were included in the test, as shown in Table 1. The experimental procedure can be summarized as follows: (1) crack 1 was first made with different depth between PZT1 and PZT2; damage detection was performed for cases OC1–OC6; (2) In addition to crack 1, crack 2 was made between PZT2–PZT3 with different depth in addition to existing crack 1; damage detection was performed for cases OC7–OC12; (3) In addition to crack 1 and crack 2, crack 3 was made between PZT3 and PZT4; damage detection was performed for cases OC13–OC18; (3) In addition to crack 1, crack 2, and crack 3, crack4 was made between PZT4 and PZT5; damage detection was performed for cases OC19–OC24.

## 4. Experimental Results and Analysis

### 4.1. Received Time-Domain Signals

**Figure 4:**
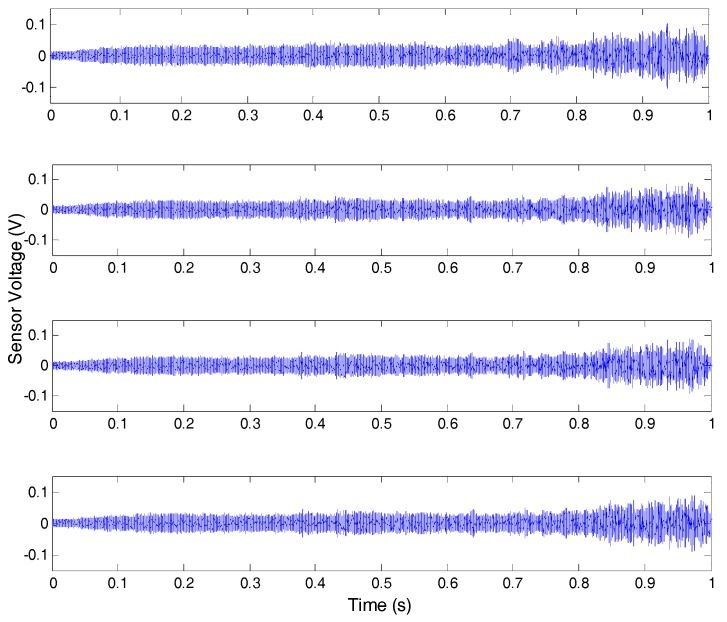
Received time-domain signals for damage case OC4.

Figure 4 demonstrates one of the test results under OC4. In this test, PZT-1 was used as the actuator, and the other PZT transducers were used as sensors. It can be seen from the experimental results that the time domain signal of PZT sensors provides very little quantitative information to evaluate crack characteristics.

### 4.2. The Damage Identification of a Single Crack in Pipeline

**Figure 5:**
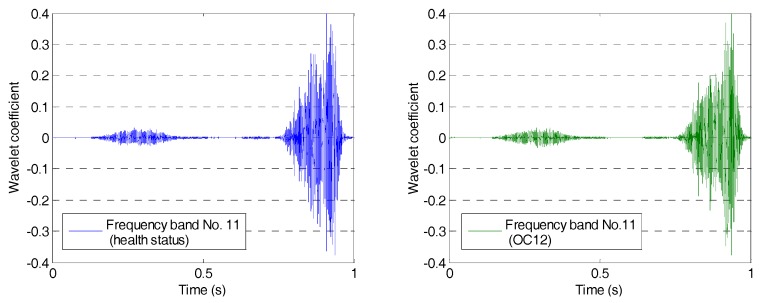
Wavelet packet coefficients comparison at frequency band No. 11.

**Figure 6:**
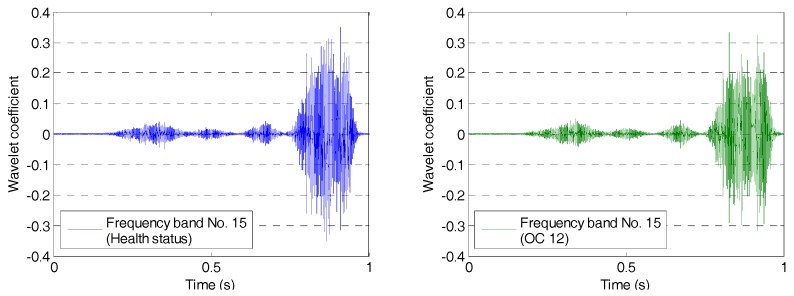
Wavelet packet coefficients comparison at frequency band No. 15.

**Figure 7:**
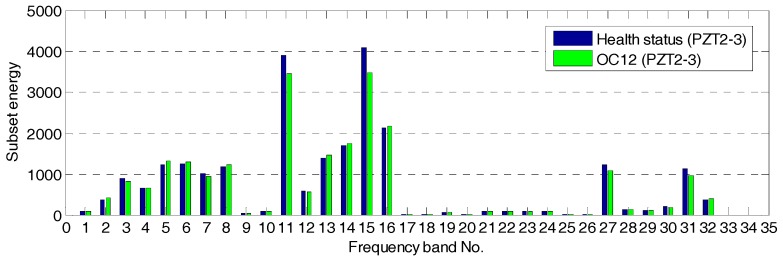
Energy vector comparison between the health status and operation condition 12 (OC12).

The wavelet packet method offers a richer signal analysis, compared with wavelet analysis results. In the wavelet analysis, the source signals are split into approximation coefficients and detail coefficients. In the next step, the approximation coefficients are split into the next level approximation coefficients and the next level detail coefficients. Successive details are never re-analyzed in wavelet analysis. However, in the wavelet packet analysis, each detail coefficient vector is also decomposed into two parts using the same approach as in approximation vector splitting. This offers a complete binary decomposition tree, which provides richer analysis. From the wavelet packet coefficients comparisons (frequency band 11) between the health status and OC12 as shown in Figure 5, the signal difference can be observed. From the wavelet packet coefficients comparisons (frequency band 15) shown in Figure 6, the obvious signal difference can also be observed in the results. An energy vector is formed by calculating the energy of each subset (frequency band) to demonstrate the energy distribution along the frequency bands. From the energy vector comparison between the healthy state and that of OC12 (as shown in Figure 7), an obvious difference can be found in different frequency bands. The proposed damage index is obtained by calculating the root-mean-square deviation between the energy vector of the health status and that of the damage status. The proposed damage index matrix is formed by damage indices of different actuator–sensor pairs at different damage cases, which represents damage situations associated with different locations and different damage severities.

**Figure 8:**
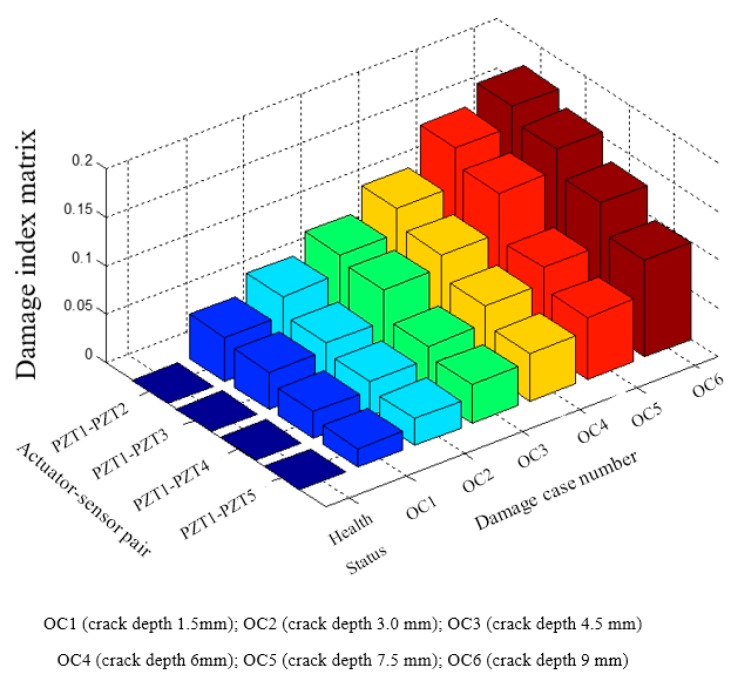
The damage index matrix for the damage cases of the first crack under different crack depths.

**Figure 9:**
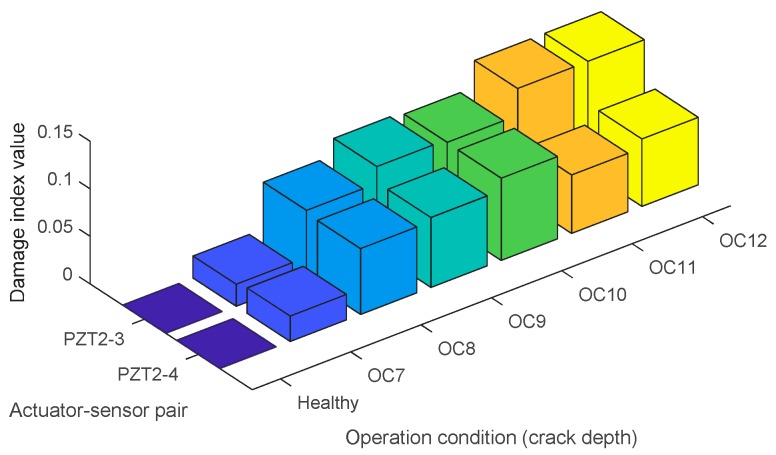
The damage index matrix for the second crack under different crack depths.

The damage index matrix of the first crack (Crack 1) under different crack depths is shown in Figure 8. In the presented damage index matrix, PZT-1 was used as the actuator and the other PZT transducers were used as sensors. The following observations can be drawn from the experimental results: (1) With the increase of the crack depth, the values of the damage indices for each sensor gradually increase. The results validate the feasibility of the proposed method in monitoring the pipeline crack severity. (2) Since PZT-2 is the closest sensor to the crack compared with other PZT sensors, the values of damage indices for PZT-2 are greater than others for the same crack depth. In addition, the values decrease from actuator–sensor pair PZT1–PZT-2 to PZT1–PZT-5 with the same crack depth, which implies that the sensitivity of the proposed method decreases with the increment of the distance between the crack and the deployed sensors. The damage index matrix of the second crack under different depths is shown in Figure 9. Crack 2 is located between PZT2 and PZT3. From OC7 to OC12, the crack depth increased from 1.5 mm to 9.0 mm. From the damage index values shown in Figure 9, it can be seen that the damage values gradually increased with the increment of crack depth of crack 2.

### 4.3. Crack Location Identification

**Figure 10:**
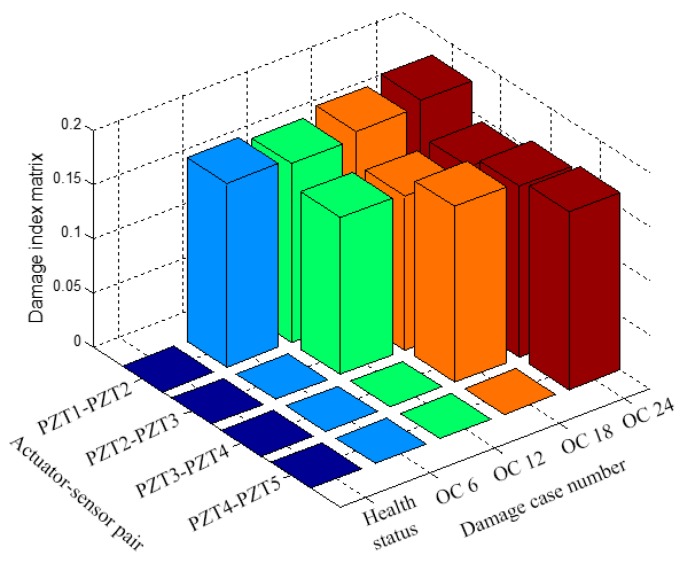
Detection of the crack’s location based on damage index matrix.

Figure 10 shows the results of using damage index matrix to identify the crack location in the pipeline structure. As shown in Figure 10, when PZT-1 was used as an actuator and PZT-2 was used as a sensor, the damage indices from four damage cases (OC6, OC12, OC18, and OC24) were presented in the given damage index matrix. Since Crack 1 was located between PZT-1 and PZT-2, an obvious increase of damage index value can be observed from the case of health status to the case of OC6. Among the damage cases from OC6 to OC24, the damage index value of PZT1–PZT2 actuator–sensor pair remains a large value due to the existence of Crack 1. For PZT2–PZT3 actuator–sensor pair, there was an obvious increase of damage index value from case OC6 to case OC12 which corresponds to those damage cases that Crack 2 was cut in addition to the existing Crack 1. Crack 1 was not located on the wave propagation path between PZT2 and PZT3; therefore, Crack 1 did not affect the damage indices of actuator–sensor pair PZT2–PZT3 for different damage cases. A similar trend can be found for the other damage indices of actuator–sensor pair PZT3–PZT4 and actuator-sensor pair PZT4–PZT5. From the damage index matrices, the location information can be extracted from damage index values of different actuator–sensor pairs. By using different actuator–sensor pairs, the proposed damage detection approach has the potential to estimate the crack location in the pipeline structure. 

## 5. Conclusions

In this paper, a feasibility study was conducted to evaluate the multiple crack damages in the pipeline structure using distributive surface-mounted PZT transducers, and a wavelet packet-based damage index matrix is proposed to evaluate pipeline integrity. Based on the experimental results, the following conclusions can be drawn:

(1) Existing cracks in the pipeline structure will induce stress wave attenuation in the wave propagation path. The attenuation ratio of propagation wave energy correlates with the crack severity. Experimental results show that the proposed damage index matrix is capable of quantitative evaluation of the crack severity at multiple locations for the pipeline structure.
(2) By using different actuator–sensor pairs of the distributive PZT transducers for health monitoring, useful crack location information can be extracted.
(3) The sensitivity of the proposed method is affected by the distance between the crack and the deployed PZT sensors.

The proposed piezoceramic-based damage detection approach is experimentally proved to be effective in the feasibility study to evaluate the crack severity and to extract the useful location information through the proposed wavelet packet-based damage index matrix. The proposed health monitoring approach has great potential to be implemented in industry to ensure pipeline structure integrity. In the authors’ future work, the large-scale pipeline structure will be tested to verify the accuracy of the proposed approach. In addition, more piezoceramic transducers will be deployed on the surface of a pipeline to study the damages in two or three dimensions.

## Figures and Tables

**Table 1:** Damage cases (experimental operating conditions).

Operating Condition	OC1	OC2	OC3	OC4	OC5	OC6
The depth of the first crack	1.5 mm	3.0 mm	4.5 mm	6.0 mm	7.5 mm	9.0 mm
Operating Condition	OC7	OC8	OC9	OC10	OC11	OC12
The depth of the second crack	1.5 mm	3.0 mm	4.5 mm	6.0 mm	7.5 mm	9.0 mm
Operating Condition	OC13	OC14	OC15	OC16	OC17	OC18
The depth of the third crack	1.5 mm	3.0 mm	4.5 mm	6.0 mm	7.5 mm	9.0 mm
Operating Condition	OC19	OC20	OC21	OC22	OC23	OC24
The depth of the fourth crack	1.5 mm	3.0 mm	4.5 mm	6.0 mm	7.5 mm	9.0 mm

Note: the state of healthy pipeline has no crack, this condition is denoted by 0.

**Table 2:** Properties of PZT-5H.

Density (g/cm^3^)	Dielectric Constant	Electromechanical Coupling Coefficient	Capacitance (nF)	Piezoelectric Coefficient (C/N)
7.50	1600	0.65	3.77	450
